# Mutant SOD1 Increases APP Expression and Phosphorylation in Cellular and Animal Models of ALS

**DOI:** 10.1371/journal.pone.0143420

**Published:** 2015-11-24

**Authors:** Polina Rabinovich-Toidman, Inna Rabinovich-Nikitin, Assaf Ezra, Beka Barbiro, Hilla Fogel, Inna Slutsky, Beka Solomon

**Affiliations:** 1 Department of Molecular Microbiology & Biotechnology, George S. Wise Faculty of Life Sciences, Tel-Aviv University,Tel-Aviv, Israel; 2 Department of Physiology and Pharmacology, Sackler Faculty of Medicine; Sagol School of Neuroscience, Tel Aviv University, Tel Aviv, Israel; Torrey Pines Institute for Molecular Studies, UNITED STATES

## Abstract

Amyotrophic lateral sclerosis (ALS) is a fatal neurodegenerative disease and it is the most common adult onset neurodegenerative disorder affecting motor neurons. There is currently no effective treatment for ALS and our understanding of the pathological mechanism is still far away from prevention and/or treatment of this devastating disease. Amyloid precursor protein (APP) is a transmembrane protein that undergoes processing either by β-secretase or α-secretase, followed by γ-secretase. In the present study, we show that APP levels, and aberrant phosphorylation, which is associated with enhanced β-secretase cleavage, are increased in SOD1^G93A^ ALS mouse model. Fluorescence resonance energy transfer (FRET) analysis suggests a close interaction between SOD1 and APP at hippocampal synapses. Notably, SOD1^G93A^ mutation induces APP-SOD1 conformational changes, indicating a crosstalk between these two signaling proteins. Inhibition of APP processing via monoclonal antibody called BBS that blocks APP β-secretase cleavage site, resulted in reduction of mutant SOD1^G93A^ levels in animal and cellular models of ALS, significantly prolonged life span of SOD1^G93A^ mice and diminished inflammation. Beyond its effect on toxic mutant SOD1^G93A^, BBS treatment resulted in a reduction in the levels of APP, its processing product soluble APPβ and pro-apoptotic p53. This study demonstrates that APP and its processing products contribute to ALS pathology through several different pathways; thus BBS antibody could be a promising neuroprotective strategy for treatment of this disease.

## Introduction

Amyotrophic lateral sclerosis (ALS) is a fatal neurodegenerative disease caused by degeneration of both upper and lower motor neurons, leading to muscle denervation and atrophy[1]. Sporadic ALS accounts for close to 90% of all ALS cases and the remaining 10% are considered familial cases, about 20% of which are caused by a dominant mutation in the gene encoding superoxide dismutase 1 (SOD1)[2]. Mutant SOD1 murine models mimic many of the clinical symptoms and pathological processes in ALS patients and have become a central research tool in discovering new pathological pathways involved in ALS[3]. Despite extensive research and new discoveries, the factors that trigger motor neuron degeneration in ALS still remain unknown.

According to recent reports, ALS patients have increased levels of amyloid precursor protein (APP) and its cleavage products, indicating their possible involvement in this disease.

APP is a type-I transmembrane protein with N-terminal extracellular and C- terminal cytoplasmic domains that belongs to the evolutionarily conserved APP protein family[4]. The role of APP in normal central nervous system (CNS) functioning is not fully understood; however previous data support its involvement in neurite outgrowth and synaptogenesis, neuronal protein trafficking along the axon, transmembrane signal transduction, cell adhesion and calcium metabolism[5]. Over-expression of APP was found in aged motor neurons, in developing spinal motor neurons undergoing programmed cell death, as well as in damaged or injured neurons[6,7]. Up-regulated APP levels were detected in the spinal cords and in the muscles of ALS patients[8,9]. Crossing of APP knockout mice with SOD1^G93A^ mice resulted in delayed motor function and body mass decline as well as improved innervation, muscle contractile characteristics, but not increased survival, suggesting that modulation, but not total depletion of APP might be beneficial in ALS[10].

Cellular APP metabolism includes processing by β-secretase cleaving enzyme (BACE1) or by α-secretase followed by γ-secretase [11]. APP cleavage products are involved in cytotoxic pathways and could contribute to pathological processes leading to neurodegeneration in Alzheimer's disease (AD) [12–14].

Here we measured APP expression, phosphorylation and processing throughout the disease progression in SOD1^G93A^ mice. Furthermore, we investigated how modulating APP processing and expression, by monoclonal antibody (MAb) that blocks the BACE cleavage site on its APP substrate (BBS), affects the progression of pathology in SOD1^G93A^ ALS mouse and cellular models. Fluorescence resonance energy transfer (FRET) analysis suggests a close interaction between SOD1 and APP at hippocampal synapses supporting that, SOD1^G93A^ mutation induces APP-SOD1 conformational changes.

## Materials and Methods

### Transgenic mice and antibody treatment

All animal experiments were conducted in accordance with the Guide for the Care and Use of Laboratory Animals and were approved by the Institutional Animal Care and Use Committee of Tel Aviv University (Permit Number: L11-017, M-15-044). All surgeries were performed under Ketamine and Xylazine anesthesia, and all efforts were made to minimize suffering.

HemizygousB6SJLTgN(SOD1G93A)1Gurmice that harbor the high copy number of the mutant allele human SOD1were obtained from the Jackson Laboratory (Bar Harbor, ME, USA). The animals were housed in standard conditions: constant temperature (22±1°C), humidity (relative, 40%), and a 12-h light/dark cycle and were allowed free access to food and water.

For evaluation of APP expression, phosphorylation and processing Tg mice and non transgenic (NT) littermates were sacrificed at different ages by intraperitonial(i.p.) anesthesia (100 mg/kg Ketamine and 20mg/kg Xylazine) following trans-cardial perfusion with saline.

Male SOD1^G93A^ mice were treated with BBS MAb or isotype-matched control non relevant anti-*Streptococcus pneumonia* MAb (non relevant MAb). The 55-days old mice were anesthetized and implanted with brain infusion cannula into the right lateral ventrical (l-2mm [Bregma] in the anterio-posterior direction, 2.8mm in mediolateral direction and 3mm depth). Brain infusion cannula was connected with osmotic mini-pump Model 2006 (Alzet, Cupertino, CA, USA), which continuously delivered the MAb (concentration 1.5 mg/mL, pumping rate 0.15 μL /h). This dosage was selected based on our previous work in AD mouse model [15]. After 42 days of treatment mice were sacrificed for biochemical (n = 4 in BBS group, n = 5 in non relevant antibody group) and histological analysis (n = 5 in BBS group, n = 5 in non relevant antibody group). Additionally, 55-day-old mice were weekly i.p. injected with 3mg/kg MAb BBS, while control mice were i.p. injected with PBS. This dosage was selected based on previously published data[16]. Disease progression, survival and behavioral analysis were performed on 48 mice. 11 i.p. treated mice were sacrificed at the age of 110 for biochemical analysis of the spinal cords (n = 6 in BBS group, n = 5 in control group).

### Clinical assessment

During the treatment period mice were weighed and their muscle strength and coordination deficits were evaluated weekly using a Rotarod apparatus (rotating at an increasing speed ranging from 4 to 40 RPM, with a constant acceleration of 1RPM per 10sec, 3 attempts). Mice were trained on the machine for 3 days before the actual beginning of the analysis. The time each mouse remained on the drum was recorded, up to 300sec. Disease onset was determined as the time animals reached their maximum bodyweight. The humane endpoint used in our experiments was defined as the inability of the mice to right themselves 30 seconds after being placed on one of their sides. Mice that met this criteria were euthanized withCO_2_. When first signs of hind limb paralysis appeared, mice health was monitored daily, and they were provided with wet mashed food in their cages.

### Tissue fractionation

Mice were sacrificed at the age of 97 or 110 days. Their spinal cords were collected and homogenized on ice in 5 volumes (w/v) of T-per extraction buffer (Pierce, USA) complemented with protease inhibitor tablets (Complete Mini Protease Inhibitor Tablets, Roche) and phosphatase inhibitor cocktail tablets (phosSTOP, Roche). After sonication homogenates were centrifuged at 100,000g for 1hr at 4°C. The resulting supernatants represent the soluble fraction.

The pellets were resuspended in T-per extraction buffer, containing protease and phosphatase inhibitors, 0.5% TritonX- 100, 1% sodium deoxycholate and 3% SDS, sonicated and centrifuged at 20,000g for 10 min at 4°C. The resulting supernatants represent the membrane fraction.

### Western blot analysis

Protein concentration was determined using BCA protein assay kit (Thermo, USA), and samples were analyzed by immunoblotting with the following primary antibodies: mouse anti-APP 22C11 (1:2000 Millipore), rabbit anti-Phospho-APP T668 (1:1000 Cell Signaling Technology), rabbit anti-sAPPβ (1:500 Covance), anti-BACE C-terminus clone 61-3E7 (Millipore), PAb-240 mouse anti p53 (1:1000 Abcam), and SD-G6 mouse anti-human SOD1 (1:1000 Sigma), mouse anti βActin (1:10000 sigma), mouse anti Tubulin (1:10000 sigma) and Mouse anti-HA antibody (1:1000 Santa Cruz). Immunoblots were developed with EZ- ECL detection kit (Biological Industries, Israel), and quantitative densitometric analysis was performed using the densitometry software EZQuant-Gel 2.12.

### Immunohistochemistry

Spinal cords collected from 97 days old SOD1^G93A^ mice were fixed in 4% (w/v) paraformaldehyde in PBS (pH 7.4) and sedimented in 30% sucrose in PBS. 30μm free-floating cryosections were prepared from lumber spinal cords. Sections were blocked with Ultra-V block (Lab Vision, USA) for 10 min. For astrocytosis evaluation sections were immunostained with rabbit anti GFAP antibody (1:500 Dako, Denmark) and applied for overnight incubation at 4°C. HRP-conjugated goat anti-rabbit IgG Fc antibody (1:1000 Jackson ImmunResearch) was applied for 1 hr. Sections were visualized using DAB chromogen substrate (Invitrogen, USA), dehydrated in graded alcohol, cleared in xylene and cover-slipped with enthelan (Merck, Germany). Image-J software (NIH, freeware) was used for the analysis.

### Primary astroglial cell cultures

Primary astroglial cells were isolated from brains of 3-day-old SOD1^G93A^ mouse pups as previously described [17]. After the meninges were removed, brains were homogenized using pipettes and incubated with 0.5 mL of trypsin for 10 min at 37°C. The resulting cell suspension was then washed with 25 mL of Dulbecco’s modified Eagle’s medium (DMEM) supplemented with 1 mM sodium pyruvate, 4 mM l-glutamine, 10% fetal bovine serum, 100 U/mL penicillin, 0.1 mg/mL streptomycin and 12.5 U/mLnystatin, followed by centrifugation at 1300 RPM at 4°C for 5 min. The supernatant was removed, and the wash was repeated. Finally, the suspension was seeded onto 6-well plates (2 mL per well) that were coated with poly d-lysine (PDL). After 7 days in culture the cells were treated with 13nM BBS or PBS for 24h. BBS concentration was selected based on our previous work [18] Primary astroglial cells were lysed in RIPA buffer (25 mM Tris•HCl pH 7.6, 150 mM NaCl, 1% NP-40, 1% sodium deoxycholate, 0.1% SDS) containing protease inhibitor tablets (Roche). The samples were sonicated for 10sec, incubated on ice for 15min and then centrifuged for 15min at 15000g. The supernatants were collected and analyzed using western blot analysis.

### NSC-34 cell culture

Inducible stable NSC-34 cells that overexpress human SOD1^G93A^fused with green fluorescence protein (GFP) were, kindly donated by Prof Nava Zisapel, and grown as described[19]. For induction of human SOD1^G93A^expression, the cells were incubated with doxycycline (1 μg/mL, 24 h). Then the cells were treated with 26nM BBS or PBS for 24h. BBS concentration was selected based on our previous work [18]. The experiment was terminated by Lysis in RIPA buffer. The samples were analyzed using Western blot analysis.

### CHO cells transfection with pcDNA3.1 plasmids containing wild-type or G93A-mutant hSOD1 cDNA

CHO cells stably transfected with WT human APP 751 isoform (CHOhAPP751) were kindly provided by D. Selkoe (Harvard Medical School, Boston)[20]. Cells were grown in DMEM (F-12) containing 10% FCS and 2.5 mM L-glutamine. hAPP 751-expressing cells were selected by using 1 mg/ml G-418 (Calbiochem) in the cells growing medium. The cells were transiently transfected with pcDNA3.1 plasmids containing either wild-type or G93A-mutant hSOD1 cDNA using Lipofectemine. After48 h validation of DNA expression was done by Western blot analysis and evaluation of Aβ levels in the condition media of the cells.

### Evaluation of Aβ 1–42 levels in the condition media of CHOhAPP751 cells

A specific ELISA for detection of Aβ 1–42 was performed. mAb 266 (raised against Aβ mid region, amino acids 17–28) was used as a capture antibody. 96 well plates were coated with mAb 266 (2.8μg/ml, diluted in 0.1M Na2CO3 pH 9.6), and incubated overnight at 4°C. On the next day, the plates were washed and blocked with 3% BSA overnight at 4°C. CHO condition media samples were collected 48 hours after transfection and incubated overnight at 4°C. On the next day, plates were washed and biotinylated mAb 22F12 (directed to Aβ 1–42 carboxy-terminus) was used as a detection antibody. 22F12 (1:2500, diluted in 1% BSA) was added to each well for a 3-hours incubation at 37°C. Bound biotinylated antibodies were detected by Extra-avidin conjugated alkaline phosphatase (diluted 1:30,000 in 1% BSA) that was incubated for 1 hour at 37°C. The dephosphorylation of p-nitrophenyl phosphate (pNPP) substrate was used to monitor Aβ levels by measuring the absorption at 405nm. The results were normalized according to number of cells.

### FRET imaging and analysis

Intensity-based FRET imaging was carried as described before [21]. CFP was excited at 440 nm and donor dequenching due to the desensitized acceptor was measured from CFP emission (460–500 nm) before and after the acceptor (YFP) photobleaching. YFP was imaged at 515 nm (excitation) and 530–560 nm (emission). Photobleaching of YFP was carried out with 515 nm laser line, by a single point activation module for rapid and efficient multi-region bleaching. Images were acquired without averaging. Image acquisition parameters were optimized for maximal signal-to-noise ratio and minimal phototoxicity. Images were 512 × 512 pixels, with a pixel width of 92–110 nm. Z-stacks were collected from 3–4 μm optical slice, at 0.6–0.8μm steps.

Mean FRET efficiency, *E*_m_, was then calculated using the equation *E*_m_ = *1 − I*_*DA*_ */I*_*D*_, where *I*_*DA*_ is the peak of donor emission in the presence of the acceptor and *I*_*D*_ is the peak after acceptor photobleaching. In order to exclude potential contribution of donor/acceptor ratio to FRET efficiency measurements, all FRET experiments were performed under saturation conditions of acceptor over donor. Detection of CFP and YFP signals was done using custom-written scripts in MATLAB as described earlier [21]. Briefly, regions of interest (ROIs) were marked at synapses that underwent YFP photobleaching. Average intensity of ROI was subtracted from background ROI intensity in close proximity to the synapse. All the ROIs that exhibited YFP photobleaching by >90% of initial fluorescence intensity were included in the analysis. Non-bleached ROIs at the same image area were analyzed to ensure lack of non-specific photobleaching due to image acquisition.

### Statistical analyses

Survival was analyzed using the Kaplan–Meier with log-rank test. Behavioral analysis, immunohistochemistry, ELISA and biochemistry, presented as the mean ± SEM, was subjected to one-way ANOVA followed by Tukey–Kramer post hoc test or Student's t-test. The critical P value was set to 0.05 for all statistical analyses.

## Results

### Effect of mutant SOD1 ^G93A^ on expression and phosphorylation of APP

**Fig 1:**
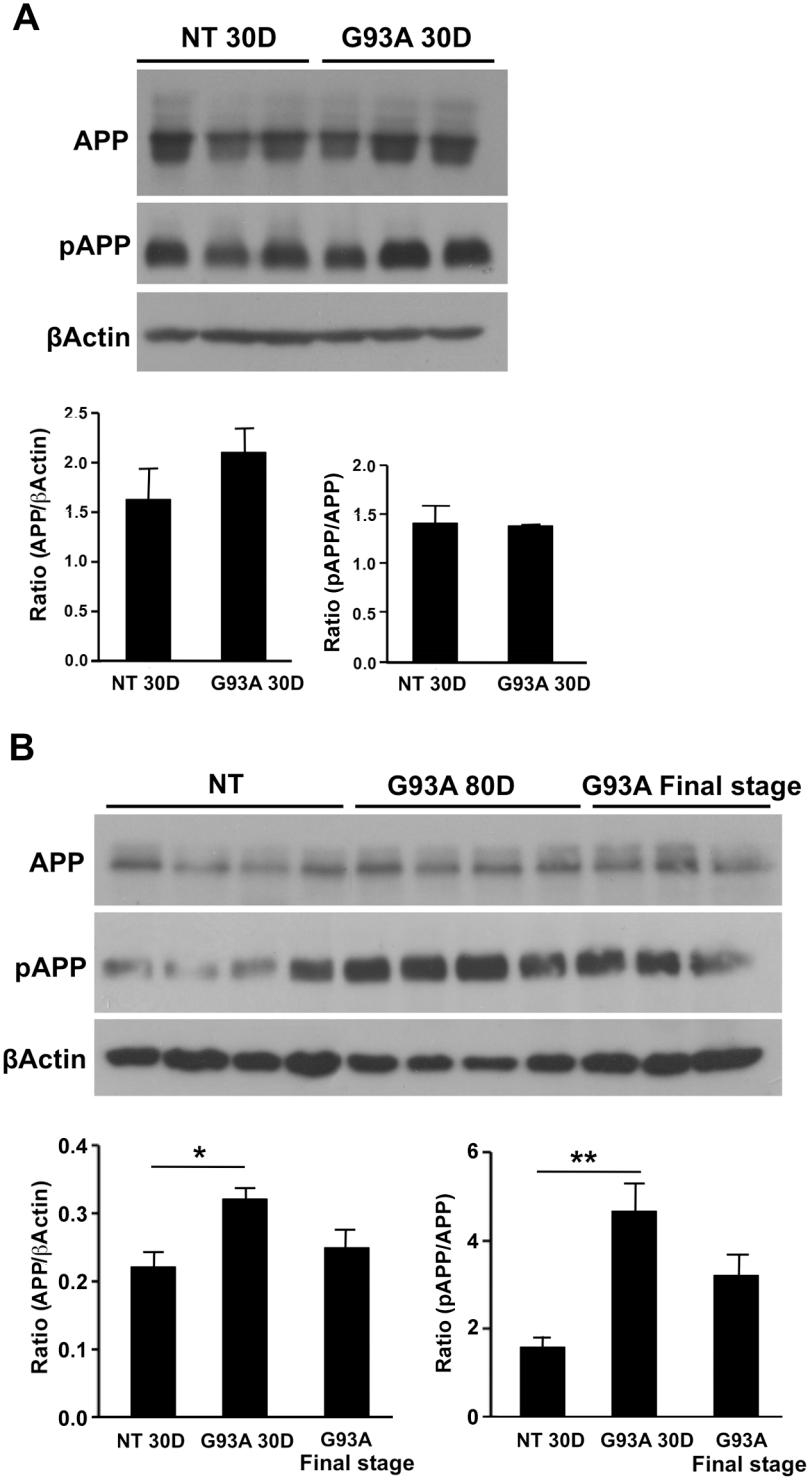
APP expression and phosphorylation in spinal cords of pre-symptomatic and symptomatic mice. Spinal cords from 30 -and 80-day-old and end stage SOD1^G93A^ mice were homogenized and their membrane fractions were subjected to immunoblot analysis. Age matched NT littermates served as control. For APP detection 22C11 antibody was used. For detection of phosphorylated APP (pAPP) a specific polyclonal anti pAPP T688 was used. β-Actin was used as an internal loading control. Immunoblot blot and densitometric analysis of the relative expression and phosphorylation levels of APP in the spinal cord of (A) 30-day-old mice, (B) 80-day-old and end stage SOD1G93A mice. All values are expressed as the mean ± SEM. Statistical comparisons were performed using one-way ANOVA followed by Tukey–Kramer post hoc test. N(NT 30D) = 3, N(G93A 30D) = 3, N(NT = 4), N(G93A 80D) = 4, N(G93A end stage = 3).*p < 0.05; **p < 0.01 (v.s. NT littermates).

The effect of mutant SOD1 on APP expression was evaluated throughout the disease progression in SOD1^G93A^ ALS mouse model. Changes in APP protein expression in the spinal cords were assessed at presymptomatic (30-day-old and 80-day-old) and symptomatic end stage of the disease compared to non Tg (NT) littermates. An increase (1.39-fold) in APP levels was detectable as early as 30 days in the spinal cords of SOD1^G93A^ mice compared to NT mice (Fig 1A). Similar trend was observed at the age of 80 days (significant 1.45-fold increase). However, at the end stage of the disease there was a non-significant reduction in the levels of APP in comparison to the levels of 80-day-old mice (Fig 1B). APP undergoes post-translational modifications such as phosphorylation at threonine 668 (T668), which is associated with enhanced BACE cleavage of APP [22]. A significant (2.9-fold increase) in T668 phosphorylation was detected in spinal cords of 80-days-old SOD1^G93A^mice (Fig 1B). In 30-days-old SOD1^G93A^ mice, there was no change in the phosphorylation of APP compared to NT mice (Fig 1A).

### Effect of mutant SOD1^G93A^ on APP processing *in vivo* and *in vitro*

**Fig 2:**
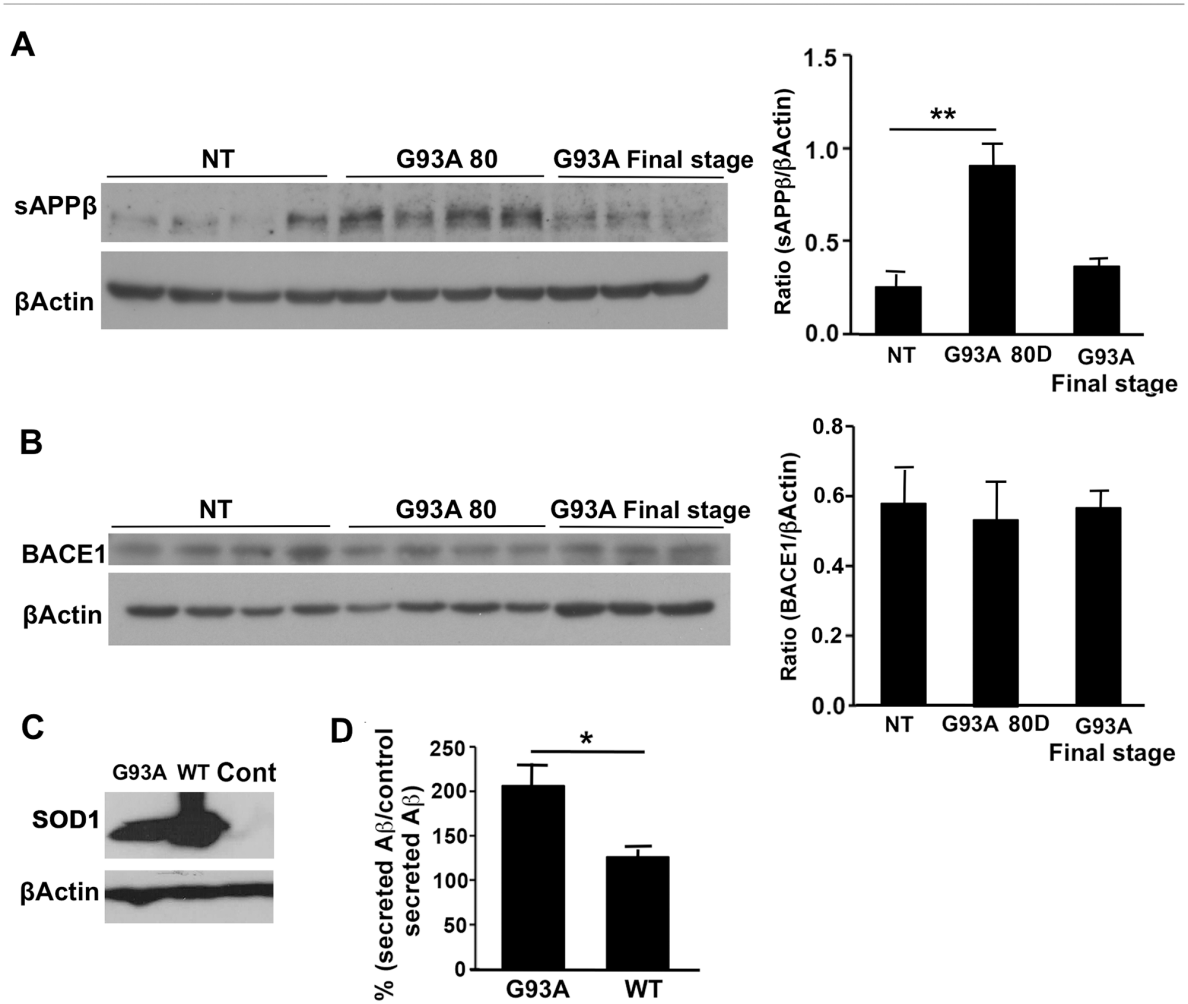
Mutant SOD1 leads to enhanced APP processing *in vivo* and *in vitro*. Soluble and membrane fractions from 80-day-old and end stage SOD1^G93A^ mice were subjected to immunoblot analysis. Age matched NT littermates served as control. A. sAPPβ was detected using polyclonal antibody specific for the neoepitope generated after BACE cleavage in the spinal cords soluble fractions. The levels of sAPPβ were normalized to β-Actin levels. B. Detection of BACE1 in the spinal cord homogenates membrane fraction was performed using a specific anti C terminal MAb. Spinal cord BACE1 levels were normalized to β-Actin levels. All values are expressed as the mean ± SEM. Statistical comparisons were performed using one-way ANOVA followed by Tukey–Kramer post hoc test. N(NT = 4), N(G93A 80D) = 4, N(G93A Final stage = 3).*p < 0.05; **p < 0.01 (v.s. NT littermates). CHO cells overexpressing APP 751 were transiently transfected with WT SOD1 or with mutant SOD1^G93A^. C. Representative Western blot of mutant and WT SOD1 expression in CHO cells as a result of transient transfection. Human HA -tagged SOD1 was detected using anti HA antibody. Non-tranfected cells served as negative control D. Aβ42 peptides levels were measured in the medium using sandwich ELISA and the ratio between secreted Aβ42 in each treatment group and the untreated group was calculated and presented in percentage. The experiment was repeated three times. Statistical comparisons were performed using Student's t-test. *p value <0.05.

We showed that APP phosphorylation was upregulated in spinal cords of SOD1^G93A^ mice. Since phosphorylation at T668 is associated with enhanced BACE processing, the levels of soluble APPβ fragment (sAPPβ), a product generated by BACE cleavage, were measured in the spinal cords of SOD1^G93A^ mice. Levels of sAPPβ were significantly elevated (3.5-fold) in 80-day-old SOD1^G93A^ mice compared to NT mice. As the disease progressed, sAPPβ levels decreased compared to pre-symptomatic 80-day-old mice (Fig 2A). This pattern correlates with expression and phosphorylation of APP throughout the different stages of the disease in the spinal cords. The increase in spinal cord sAPPβ levels is not attributed to increased BACE1 expression, since there was no change in BACE1 levels at any stage of the disease, in comparison to NT mice (Fig 2B). The effect of mutant SOD1^G93A^ on APP processing in vitro was demonstrated in Chinese hamster ovary (CHO) cells overexpressing human APP 751. The cells were transiently transfected with either HA -tagged WT SOD1 or SOD1^G93A^. As shown in Fig 2C, HA -tagged SOD1 was expressed only in transfected cells. Extracellular Aβ42 levels were measured in the medium two days after transfection using sandwich ELISA. Fig 2D shows a significant 1.6-fold increase in secreted Aβ42 levels in the condition media of cells transfected with mutant SOD1 compared to cells that were transfected with WT SOD1.

### BBS treatment leads to reduction in APP and SOD1^G93A^ levels

**Fig 3:**
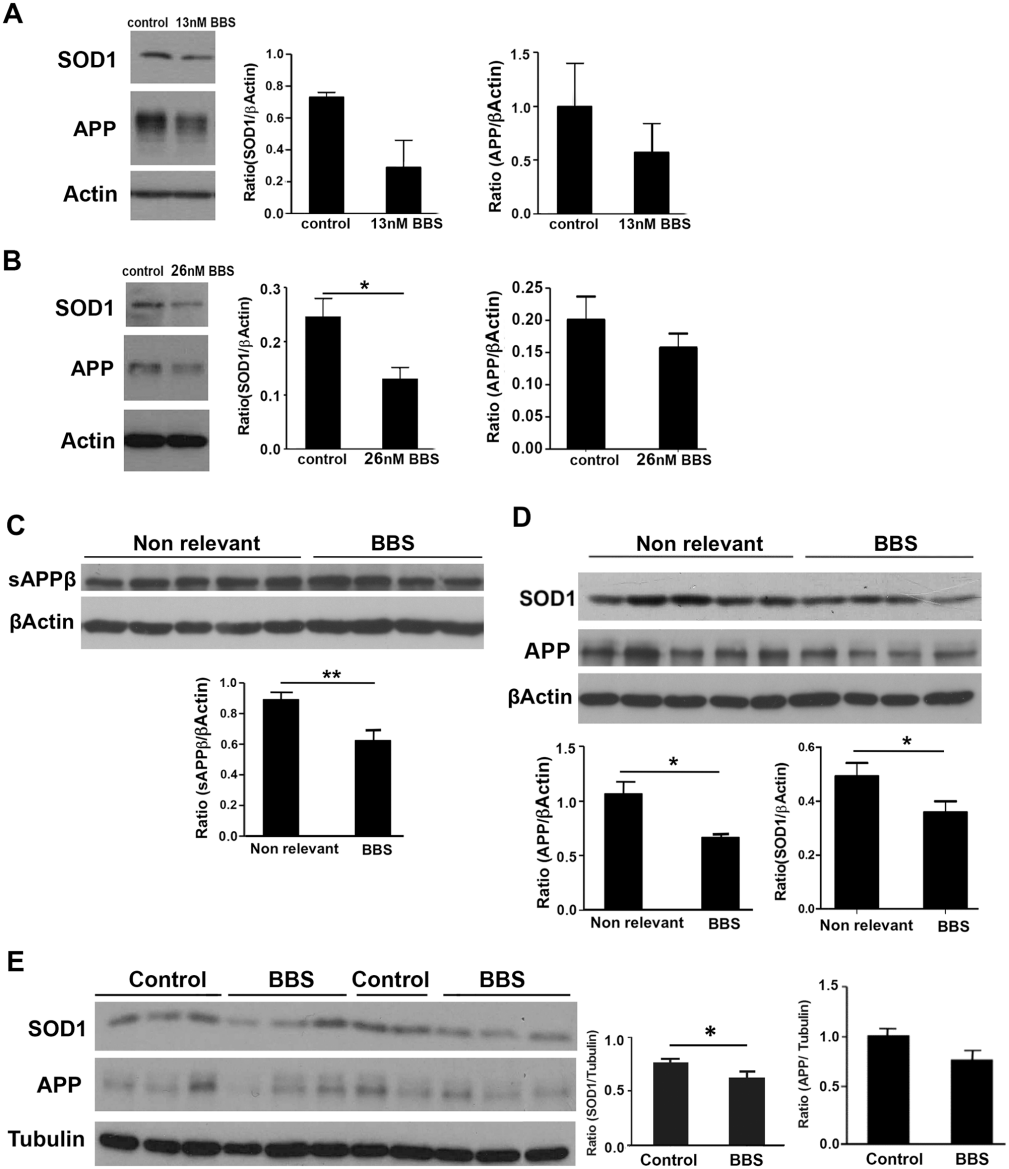
BBS treatment results in reduction of APP and mutant SOD1^G93A^ levels *in vitro* and *in vivo*. Primary astroglial cells were isolated from brains of mutant SOD1^G93A^ pups. After 7 days in culture cells were treated with 13nM BBS for 24h. Induction of mutant SOD1^G93A^ fused to GFP expression in NSC34 stable line was performed by incubating the cells with Doxycycline for 48h. 24h after the induction the cells were treated with 26nM BBS for 24h. The levels of mutant SOD1, APP and actin were measured using Western blot analysis. SOD1 was detected by polyclonal anti human SOD1 antibody and APP was detected using 22C11 antibody. SOD1 levels were normalized to β-Actin levels. A. Levels of APP and SOD1 in primary astroglial cells. B. Levels of APP and SOD1 fused to GFP in NSC34 stable line. All values are expressed as the mean ± SEM. Statistical comparisons were performed using Student's t-test. *p < 0.05. For in *vivo* analysis of the effect of BBS, spinal cords of i.c.v. and i.p. treated SOD^G93A^ mice were homogenized and subjected to immunoblot analysis. Analysis of the effect of BBS, in spinal cords of i.c.v. treated SOD^G93A^ mice C. sAPPβ was detected in spinal cord soluble fractions and normalized to β-Actin levels. D. APP and SOD1 in spinal cords of i.c.v. treated mice were detected in membrane fractions and normalized to β-Actin. All values are expressed as the mean ± SEM. Statistical comparisons were performed using Student's t-test. N(BBS) = 4, N(Non relevant) = 5. *p < 0.05; **p < 0.01 (v.s. Non relevant). Analysis of the effect of BBS, in spinal cords of i.p. treated SODG93A mice. E. The levels of mutant SOD1, APP and tubulin in the membrane fraction were measured using Western blot analysis. Statistical comparisons were performed using Student's t-test. N(BBS) = 12, N(Control) = 11.*p < 0.05.

The increase in APP expression, phosphorylation and processing in ALS mice model, indicates that there might be a crosstalk between APP and mutant SOD1. To explore this possibility APP processing was modulated using BBS MAb. Cellular models including primary astroglial cells isolated from SOD1^G93A^ mice and inducible NSC34 stable line overexpressing mutant SOD1^G93A^were treated with BBS. The treatment led to decrease in the levels of APP in primary astroglial cells (1.7-fold) and in NSC34 SOD1^G93A^ cells (1.2-fold) (Fig 3A and 3B). Mutant SOD1 levels were also reduced as a result of the treatment in both astrocytes (2.5-fold) and NSC34 SOD1^G93A^ cells (1.86-fold) (Fig 3A and 3B). The effect of BBS was also investigated in 55-days-old SOD1^G93A^ mice treated with the MAb intraventricularly (i.c.v.). Control group received the same dose of non-relevant MAb. sAPPβ (1.4-fold), APP (1.5-fold) and SOD1^G93A^ (1.37-fold) levels were significantly reduced in spinal cords of BBS treated mice (Fig 3C and 3D). To further support reduction in APP processing by BBS treatment in the CNS, sAPPβ and APP levels were also measured in brain homogenates. Western blot analysis showed a similar reduction in APP and sAPPβ levels in the brain (S1 Fig). We also investigated the effect of systemic treatment on the levels of APP and mutant SOD1. Female and male mice received weekly i.p. injection of 3mg/kg BBS starting from the age of 55 days. The control group was injected with PBS. They were sacrificed at the age of 110 days. Similar to the above data, the treatment resulted in reduction in the levels of APP (1.2 fold) and mutant SOD1 (1.2 fold) in the spinal cords of SOD1^G93A^ mice (Fig 3E). Manipulation of APP processing *in vivo* and *in vitro* leads to reduction of mutant SOD1 levels.

### APP and SOD1 interact at hippocampal synapses

**Fig 4:**
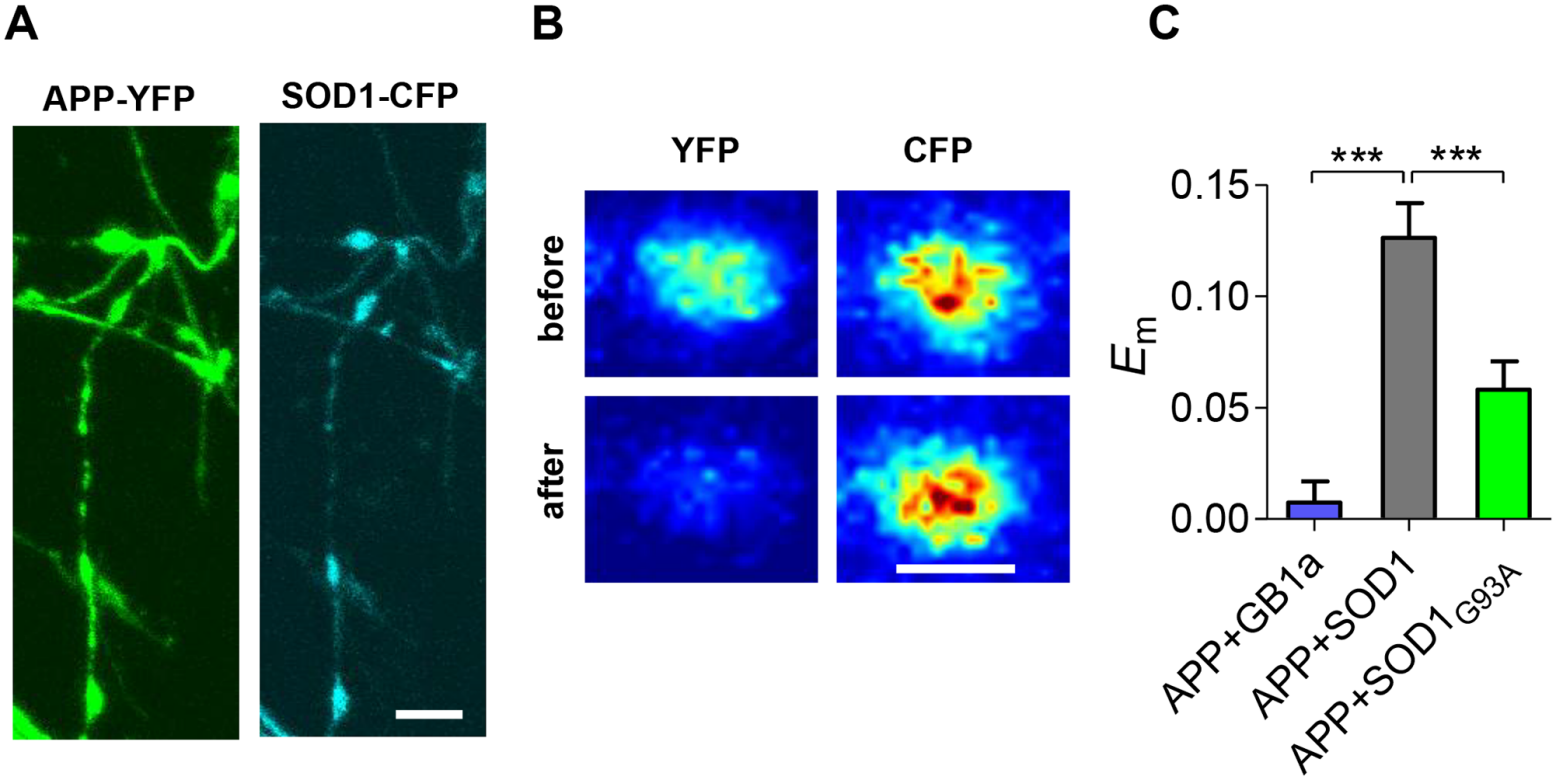
APP molecules closely associate with SOD1 at single hippocampal synapses. A. Representative confocal images of boutons of a hippocampal neuron that was co-transfected with APP^YFP^ and SOD1^CFP^. Scale bar: 2 μm. B. Pseudocolor-coded fluorescence images of a bouton expressing APPYFP and SOD1CFP before and after YFP photobleaching, Scale bar: 2 μm. C. Summary of Em data for APPYFP / GB1aCFP (n = 26), APPYFP / SOD1CFP (n = 39), APPYFP / SOD1CFP (n = 42). Error bars represent SEM.

The correlation between APP and SOD1 expression levels in ALS models indicates that these proteins may physically interact, leading to structural and functional crosstalk between their signaling processes. To examine whether SOD1 and APP are closely associated at individual hippocampal synapses, we utilized FRET spectroscopy that enables studying conformational changes between fluorescently tagged proteins [23] by providing an accurate measure of possible changes in the relative distance (<100 Å) and/or orientation between fluorophores. Hippocampal cultures were co-transfected with APP C-terminally tagged with YFP (APP^YFP^) and SOD1 C-terminally tagged with CFP (SOD1^CFP^). FRET measurements were taken from synapses of hippocampal neurons (Fig 4A). We measured the steady-state mean FRET efficiency (*E*_m_) utilizing the acceptor photobleaching method, which dequenches the donor (CFP). FRET efficiency was measured in the presence of tetrodotoxin (TTX) which blocks spike generation, allowing only miniature synaptic release. High-magnification confocal images show an increase in Cer fluorescence after Cit photobleaching (Fig 4B), indicating dequenching of the donor and the presence of FRET. Quantitative analysis of FRET signals between APP^YFP^ and SOD1^CFP^ proteins across 39 synapses from 4 experiments under miniature synaptic activity revealed a mean *E*_m_of 0.13 ± 0.02 (Fig 4C). The FRET level varied across different synapses (coefficient of variation of 77%) suggesting different APP-SOD1 conformational states at the single-synapse level. To rule out non-specific association due to over-expression of the tagged proteins, we co-transfected APP^YFP^ and a non-related GB_1a_ protein, a subunit of the GABA_B_ receptor, C-terminally tagged with CFP (GB_1a_^CFP^). These two tagged proteins showed negligible FRET (Fig 4C). Therefore, the higher FRET efficiencies measured between APP^YFP^ and SOD1^CFP^ (p < 0.001) are due to the specific association of APP^YFP^ and SOD1^CFP^ proteins at hippocampal synapses. These data indicate that there might be an interaction between SOD1 and APP under physiological conditions. Interestingly, SOD1^G93A^ mutation induced a ~54% reduction (p <0.001) in FRET efficiency between SOD1_G93A_^CFP^ and APP^YFP^ (0.06 ± 0.01, Fig 4C). These results suggest that G93A mutation in the SOD1 sequence leads to APP-SOD1 conformational changes that may contribute to ALS pathophysiology and progression.

### Beneficial effect of BBS treatment in SOD1^G93A^ mice

**Fig 5:**
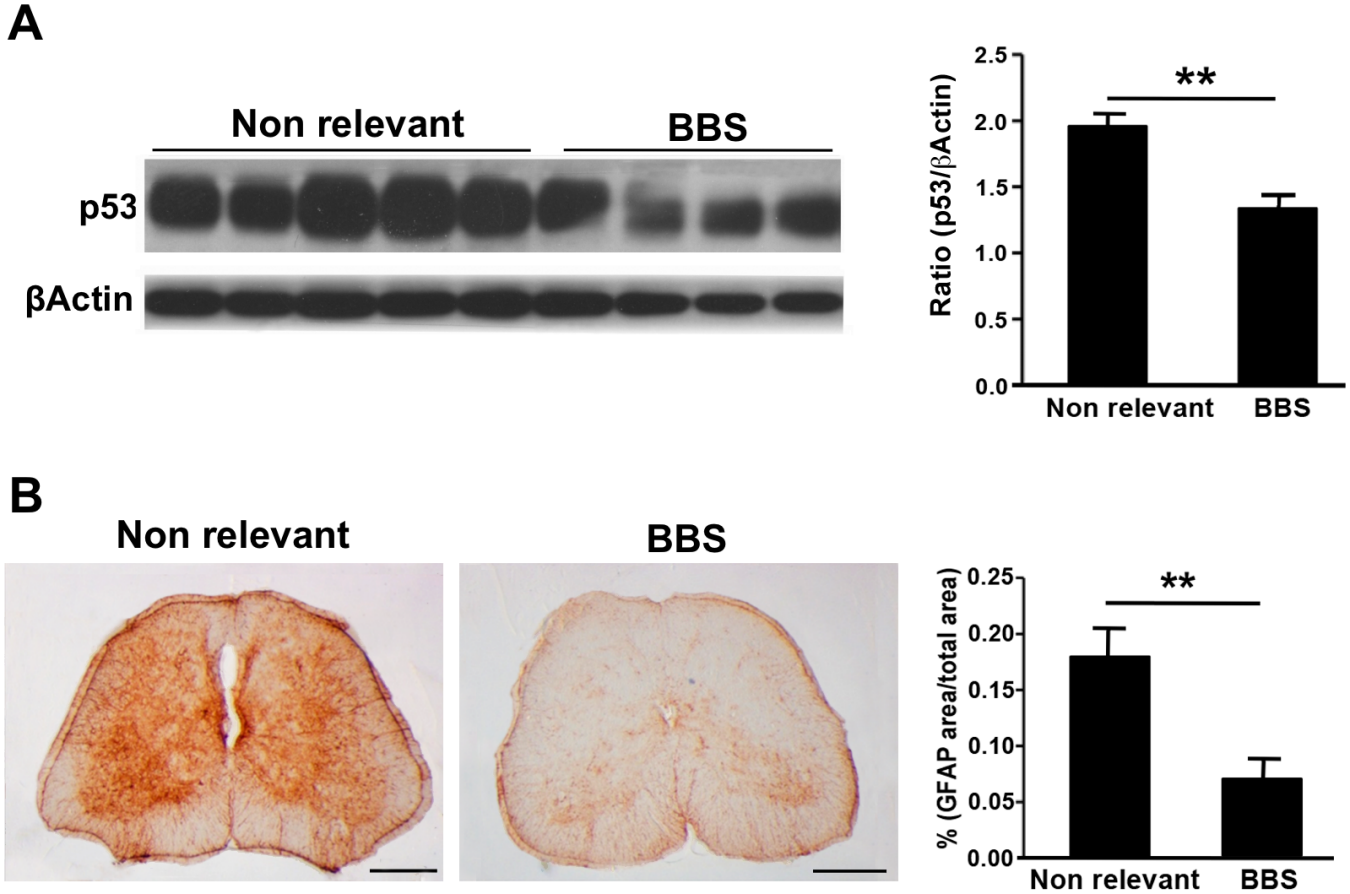
BBS treatment leads to decrease in p53 levels and astrogliosis marker GFAP. A. The levels of p53were quantified using immunoblot analysis in the soluble fraction of spinal cord homogenates of SOD^G93A^ mice treated with BBS or non relevant MAb for 42 days. p53 levels were detected using PAb 240 antibody and normalized to β-Actin. All values are expressed as the mean ± SEM. Statistical comparisons were performed using Student's t-test. N(BBS) = 4, N(Non relevant) = 5. *p < 0.05; **p < 0.01 (v.s. Non relevant). B. Lumber spinal cords were subjected to immunohistochemical analysis. Spinal cord sections were stained with anti-GFAP antibody, and the intensity of the staining was analyzed using Image-J Software. Scale bars in panel B correspond to 1 mm, respectively. Statistical comparisons were performed using Student's t-test. N(BBS) = 5, N(Non relevant) = 5. **p < 0.01 (v.s. Non relevant).

Having established an increase in APP expression, phosphorylation and processing in ALS mice, we further characterized the involvement of APP in ALS by investigating whether partial inhibition of APP processing via BBS antibodies is neuroprotective in SOD1^G93A^ mice. Effect of APP and its cleavage products might be mediated through downstream toxic targets. AICD was proposed to up-regulate transcription of pro-apoptotic p53 [12,14]. p53 might be involved in neuronal degeneration in spinal cords of SOD1^G93A^ mice [24]. Therefore the effect of BBS treatment on p53 expression was investigated by western blot analysis in spinal cord homogenates of BBS treated SOD1^G93A^ mice. We found that there was a significant 1.5-fold reduction in p53 levels in spinal cords of SOD1^G93A^ mice (Fig 5A).

To further assess the neuroprotective effect of BBS MAb, lumber spinal cords of treated SOD1^G93A^ mice were collected and subjected to immunohistochemical analysis. Since astrocytes play a major role in ALS pathology [25], and it was previously shown that BBS leads to reduction in the levels of activated astrocytes marker glial fibrillary acidic protein (GFAP), we examined the effect of BBS treatment on astrogliosis in SOD1^G93A^ mice. Lumber spinal cord sections were probed for the presence of GFAP. There was a significant 2.5-fold decrease in the levels of GFAP in the lumber spinal cords of BBS treated SOD^G93A^ mice (Fig 5B) compared to control. These data indicate that inhibition of APP processing reduces astrogliosis in SOD1^G93A^ mice.

### BBS treatment prolongs survival of SOD1^G93A^ mice

**Fig 6:**
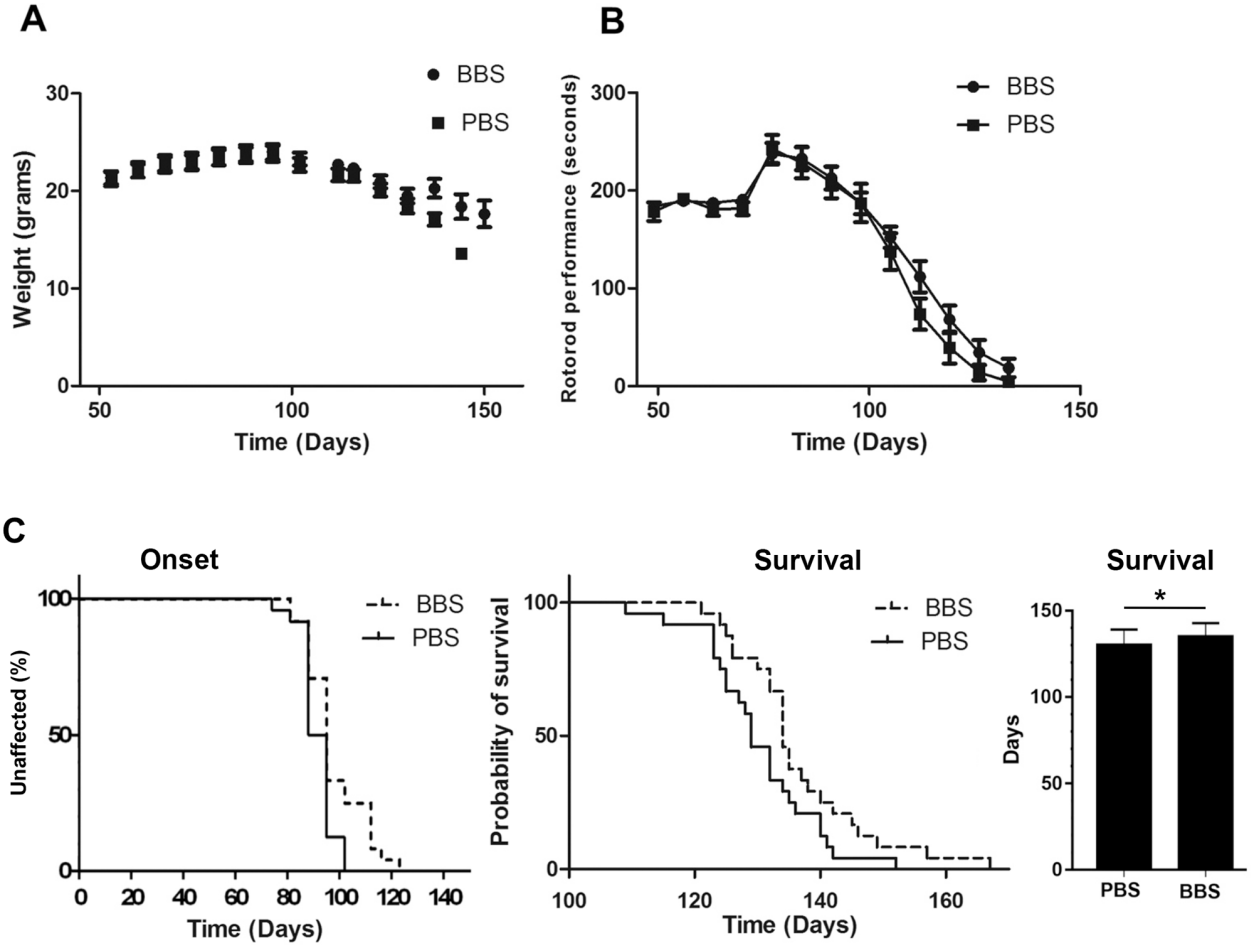
BBS treatment leads increase in survival of SOD1^G93A^ transgenic mice. 55-day-old female and male SODG93A mice received a weekly dose of 3mg/kg MAb BBS via i.p. injection. Controls were treated with PBS. A. Weight of each animal was recorded weekly. B. Motor functions of the i.p. treated mice were assessed by performing Rotarod test. Mice were trained to run on the 2-cm-diameter rod, which rotated at a fixed speed of 13 turns per minute. Mice were allowed to run for up to 1 min in each trial, or until they fell off. C. Plots of disease onset (Median BBS = 95; Median PBS = 91.5; *p <0.05; Average BBS = 97.2; Average = 91.9; *p <0.05), survival(Median BBS = 95; Median PBS = 91.5; *p <0.05; Average BBS = 97.2; Average = 91.9; *p <0.05) and the average survival in days. N (PBS) = 24, N (BBS) = 24.

Our data showed that BBS treatment led to reduction in the levels of several markers that are associated with ALS pathology. We investigated the effect of BBS treatment on disease progression and survival.55-day-old SOD1^G93A^ mice were weekly i.p injected with of 3mg/kg MAb BBS, while the controls were injected with PBS. Disease onset was defined at the age when mice reached their peak weight. Administration of BBS to SOD1^G93A^ mice delayed disease onset (Median BBS = 95; Median PBS = 91.5; *p <0.05; Average BBS = 97.2; Average = 91.9; *p <0.05) (Fig 6C). In addition there was a slight improvement in the motor function, assessed by Rotarod test. Moreover, 55-days-old BBS-treated mice had a significant 5-day extension in life span compared to control-treated mice (Median BBS = 134; Median PBS = 129; *p <0.05; Average BBS = 134.7; Average = 129.9; *p <0.05) (Fig 6C).

## Discussion

The present study demonstrates that there is a crosstalk between SOD1 and APP, two proteins that play a crucial role in neurodegeneration. We showed that APP levels, and phosphorylation at threonine 688, which is associated with enhanced β-secretase cleavage,are increased in spinal cords of presymptomatic mutant SOD1G93A mice [26]. We found that APP phosphorylation is increased only in spinal cords of 80-day-old mice, just before the clinical onset of the disease and was accompanied by elevated levels of the BACE-processing product sAPPβ. This data is further supported by the in vitro results in CHO cellular model. SOD1 overexpression in CHO cells that overexpress APP leads to increased Aβ secretion. The fact that BACE1 levels remain unchanged in spinal cords of transgenic mice suggests that increased APP processing is a result of increased APP phosphorylation. Indeed, increased phosphorylation related to processing was detected in AD patients[27]. The increased phosphorylation of APP might be a consequence of upstream pathological pathways that occur prior to disease onset. This data indicates that mutant SOD1 promotes changes in APP expression and post translational modifications that lead to increased production of APP processing products.

ALS is a motor neurons disease characterized by muscle paralysis,nonetheless45% of ALS patient have cognitive symptoms similar to AD[28]. Moreover, according to recently published research that examined ALS patient neuropathology post-mortem, pathological amyloid beta immunohistochemistry was present in ALS patients with or without neuropathological features of AD[29]. Here we show that aberrant APP phosphorylation and processing occurs not only in AD, but also in ALS mouse model. This accumulating data suggests that there might be a clinicopathological overlap between ALS and AD.

Inhibition of APP processing via MAb BBS led to reduction in the levels of APPand sAPP beta in the spinal cords and brains of SOD1^G93A^ Tg mice. Additionally, mutant SOD1^G93A^levels were decreased in spinal cords of BBS treated mice. Supporting these findings are the results in NSC34 cells and in primary astroglial cells that overexpress mutant SOD1^G93A^. As in vivo BBS treatment, in vitro treatment, resulted in reduction in APP and SOD1^G93A^ levels, indicating that through inhibition of APP processing it is possible to reduce mutant SOD1levels and possibly attenuate its toxicity. The interplay between APP and SOD1 suggests their interaction. FRET spectroscopy results indicate that both SOD1 and APP are found in a close proximity(< 10 nm) at the level of individual hippocampal synapses, suggesting that APP-SOD1 inter-molecular interaction that may play yet unknown physiological role in synaptic function. It is noteworthy that FRET efficiency between APP and mutant SOD1 is significantly lower compared to WT SOD1, indicating that G93A SOD1 mutation triggers APP-SOD1 conformational changes. Further studies are needed to understand how APP-SOD1^G93A^ conformational changes may contribute to ALS pathophysiology.

The fact that BBS treatment resulted in decrease in APP levels and not only in inhibition of its processing is surprising. One of the possible pathways that might lead to down-regulation of APP expression is reduced levels of the C-terminal APP-derived peptide AICD. AICD translocates into the nucleus where it functions as a transcription factor and induces expression of APP as well as a series of proteins involved in cell death, such as p53[12,30,31]. We found that there was a significant reduction in the levels of p53 in the spinal cords of BBS treated mice. This data further supports that AICD might mediate downregulation of protein expression in BBS treated mice. Due to AICD extreme instability we were unable to detect it in spinal cords[32]. However, we assume that BBS-mediated reduction in APP processing leads to decreased AICD levels. p53 is involved in ALS pathology; therefore it seems that inhibition of APP processing may block pro-apoptotic signaling mediated through p53 activity in ALS mice[33].

In patients and mouse models of ALS, inflammatory responses accompany motor neuron degeneration[25]. It was shown that GFAP levels were increased in APP and mutant SOD1 double Tg mice compared to mutant SOD1 single Tg mice [34]. This data indicates that increased levels of APP or its processing products lead to augmentation of inflammatory processes in ALS SOD1^G93A^ mice. We found that in correlation with reduction in APP levels and processing, BBS MAb treatment dramatically reduced GFAP levels in lumbar spinal cords. Thus APP or its processing products directly or indirectly are involved in neuroinflammation in ALS. It was previously shown that there is a significant increase in the number of activated astrocytes in spinal cords of SOD1^G93A^ mice compared to WT mice [35]. We have demonstrated that BBS treatment results in decrease in mutant SOD1 levels not only in motor neuron cell line NSC34 but also in primary astroglial cells; therefore it is possible that reduction in neuroinflammation occurs as a result of reduction in the levels of mutant SOD1.

Since upregulation of APP expression and processing occur before disease onset, we examined the effect of inhibition of APP cleavage at pre-symptomatic stage of disease in SOD1^G93A^ mice. Our previous data showed that brain infusion of BBS MAb resulted in a minor beneficial effect in symptomatic 90- day-old SOD1 mice, while earlier administration of BBS at 70 days resulted in a delay of disease onset and deterioration [36]. A greater beneficial effect was obtained when treatment started at 55 days. This data indicates that APP or its processing products are involved in early pathological changes that precede clinical onset of the disease.

## Conclusions

Our data show that APP expression, phosphorylation and processing were increased in pre-symptomatic ALS SOD1^G93A^ mice. It seems that interference with APP processing results in decrease in mutant SOD1 levels. The interaction between these two molecules occurs at synapses. Since all changes in APP expression and processing occur before onset of the disease, inhibition of APP processing is much more effective at early stages of the disease. The pathological function of APP might be mediated through production of toxic products such as AICD that regulates expression of downstream targets one of which is pro-apoptotic p53. Taken together, these findings suggest that treatment of ALS with an appropriate dose of BBS MAb at the pre-symptomatic stage could be a promising neuroprotective strategy for treatment of this debilitating disease. We conclude that APP and its processing products play a significant role in early pathological processes including neuro-inflammation, which is known for its crucial contribution to neurodegeneration in ALS.

## Supporting Information

S1 Fig BBS treatment reduced APP processing and expression in the brains of SOD^G93A^ mice. The product of βsecretase cleavage of APP called sAPPβ as well as APP were measured in the brain homogenates of i.c.v. treated SOD^G93A^ mice using Western blot analysis. A. sAPPβ was detected in the brain using polyclonal antibody specific for the neoepitope generated after βsecretase cleavage and βactin was detected using monoclonal antibody. sAPPβ levels were normalized to βactin levels. *p value for sAPPβ = 0.02. B. APP was detected using 22C11 antibody. Brain APP levels were normalized to βactin levels. *p value for brain APP = 0.01. N(BBS) = 4, N(Non relevant) = 5.
(JPG)
