# Supplementary figures and images for: Mutant SOD1 Increases APP Expression and Phosphorylation in Cellular and Animal Models of ALS
Source: PLoS One. 2015 Nov 24;10(11):e0143420. doi: 10.1371/journal.pone.0143420 (PMC4658003; doi:10.1371/journal.pone.0143420)

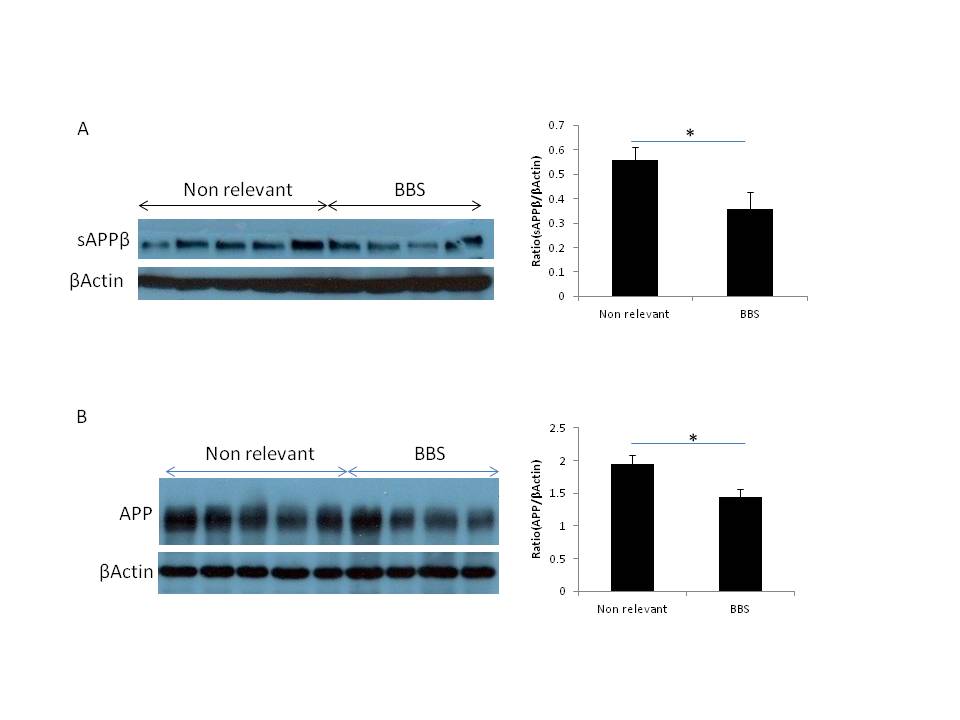

Supplement: S1 Fig — The product of βsecretase cleavage of APP called sAPPβ as well as APP were measured in the brain homogenates of i.c.v. treated SODG93A mice using Western blot analysis. A. sAPPβ was detected in the brain using polyclonal antibody specific for the neoepitope generated after βsecretase cleavage and βactin was detected using monoclonal antibody. sAPPβ levels were normalized to βactin levels. *p value for sAPPβ = 0.02. B. APP was detected using 22C11 antibody. Brain APP levels were normalized to βactin levels. *p value for brain APP = 0.01. N(BBS) = 4, N(Non relevant) = 5. (JPG) [file pone.0143420.s001.jpg]
